# Supplementary material for: Modeling glioblastoma heterogeneity as a dynamic network of cell states
Source: Mol Syst Biol. 2021 Sep 16;17(9):e10105. doi: 10.15252/msb.202010105 (PMC8444284; doi:10.15252/msb.202010105)
Supplement: Supplementary file 5 — Source Data for Figure 3 [file MSB-17-e10105-s001.zip › Figure3A_sourcedata/GSEA_3065/hallmarks_state6.GseaPreranked.1623416308702/HALLMARK_APICAL_SURFACE.html]

Details for gene set HALLMARK\_APICAL\_SURFACE[GSEA]

|  || Dataset | state6 |
| Phenotype | NoPhenotypeAvailable |
| Upregulated in class | na\_neg |
| GeneSet | HALLMARK\_APICAL\_SURFACE |
| Enrichment Score (ES) | -0.59176284 |
| Normalized Enrichment Score (NES) | -1.6746823 |
| Nominal p-value | 0.014732965 |
| FDR q-value | 0.01003572 |
| FWER p-Value | 0.086 |
Table: GSEA Results Summary

  

Fig 1: Enrichment plot: HALLMARK\_APICAL\_SURFACE      
 Profile of the Running ES Score & Positions of GeneSet Members on the Rank Ordered List

  

| PROBE | GENE SYMBOL | GENE\_TITLE | RANK IN GENE LIST | RANK METRIC SCORE | RUNNING ES | CORE ENRICHMENT || 1 | FLOT2 |  |  | 468 | 0.127 | 0.0143 | No |
| 2 | GSTM3 |  |  | 902 | 0.091 | 0.0134 | No |
| 3 | NCOA6 |  |  | 934 | 0.089 | 0.0575 | No |
| 4 | HSPB1 |  |  | 3587 | 0.010 | -0.2395 | No |
| 5 | PLAUR |  |  | 3956 | 0.001 | -0.2807 | No |
| 6 | TMEM8B |  |  | 4599 | -0.011 | -0.3482 | No |
| 7 | SHROOM2 |  |  | 4660 | -0.012 | -0.3487 | No |
| 8 | CX3CL1 |  |  | 5266 | -0.024 | -0.4051 | No |
| 9 | CRYBG1 |  |  | 5365 | -0.026 | -0.4026 | No |
| 10 | ADIPOR2 |  |  | 5514 | -0.029 | -0.4042 | No |
| 11 | B4GALT1 |  |  | 5870 | -0.036 | -0.4256 | No |
| 12 | EPHB4 |  |  | 6204 | -0.043 | -0.4408 | No |
| 13 | GAS1 |  |  | 6366 | -0.046 | -0.4344 | No |
| 14 | SULF2 |  |  | 6960 | -0.064 | -0.4681 | No |
| 15 | ADAM10 |  |  | 7615 | -0.087 | -0.4963 | No |
| 16 | THY1 |  |  | 8454 | -0.153 | -0.5103 | Yes |
| 17 | BRCA1 |  |  | 8659 | -0.217 | -0.4177 | Yes |
| 18 | DCBLD2 |  |  | 8676 | -0.226 | -0.2989 | Yes |
| 19 | SRPX |  |  | 8746 | -0.285 | -0.1548 | Yes |
| 20 | APP |  |  | 8758 | -0.300 | 0.0043 | Yes |
Table: GSEA details [plain text format]

  

Fig 2: HALLMARK\_APICAL\_SURFACE: Random ES distribution      
 Gene set null distribution of ES for **HALLMARK\_APICAL\_SURFACE**
